# Community’s perceptions of the police during COVID-19 in Harlem, New York, a predominantly Black community: Social and geographical dimensions

**DOI:** 10.1371/journal.pone.0329027

**Published:** 2025-10-06

**Authors:** Victoria K. Ngo, Thinh T. Vu, Victoria Sunseri, Srividhya Sharma, Deborah Levine, Malcolm A. Punter, Pedro Mateu-Gelabert, Luisa N. Borrell

**Affiliations:** 1 Center for Innovation in Mental Health, Graduate School of Public Health and Health Policy, The City University of New York, New York, New York, United States of America; 2 Department of Community Health and Social Sciences, Graduate School of Public Health and Health Policy, The City University of New York, New York, New York, United States of America; 3 Harlem Health Initiative, Graduate School of Public Health and Health Policy, The City University of New York, New York, New York, United States of America; 4 Harlem Congregations for Community Improvement, Inc., New York, New York, United States of America; 5 Department of Epidemiology and Biostatistics, Graduate School of Public Health and Health Policy, The City University of New York, New York, New York, United States of America; 6 Department of Surgery, Medical and Social Sciences, Faculty of Medicine and Health Sciences, Public Health and Epidemiology Research Group, Universidad de Alcalá, Alcalá de Henares, Madrid, Spain; Universitat de Girona, SPAIN

## Abstract

**Introduction:**

Numerous demonstrations protesting policing practices towards the Black community have sprung up across the U.S. in recent years, especially during COVID-19. We examined the community’s perception of police and its association with social (e.g., a history of discrimination and community safety) and geographical (e.g., housing type and district community) factors in New York City.

**Methods:**

An online survey of 393 adults in Harlem was conducted between April and September 2021. A 10-item questionnaire asked about police responsiveness, safety, and racism, with higher scores indicating more positive perception of the police. Multivariable linear regression examined the association of social and geographical factors with community perceptions of the police.

**Results:**

The mean score of community’s perception of the police was 10.33 (SD = 3.73) out of 25, indicating a generally negative view. Notably, 39% doubted police’s capacity to handle mental health issues, 36% felt police were disrespectful to demonstrators, and 35% perceived police behavior as racist. In the adjusted model, younger age groups (18−29 years old: β = −2.45, 95%CI: −3.61, −1.28; 30−39 years old: β = −1.80, 95%CI: −2.82, −0.77 vs. 40−81 years old) showed significantly less favorable perceptions of the police. Higher education (associate’s or college degree: β = −0.83, 95%CI: −1.77, −0.10; bachelor’s or graduate degree: β = −2.10, 95%CI: −3.15, −1.05 vs. high school or less) and being employed (β = −2.15, 95%CI: −3.32, −0.99 vs. unemployed) were also associated with more negative perceptions. Those living in low-income housing (β = −0.87, 95%CI: −1.62, −0.12) rather than market-rate housing and facing discrimination (β = −1.94, 95%CI: −2.72, −1.16), had more negative perceptions of the police. Interestingly, community safety during COVID-19 (β = −0.63, 95%CI: −1.05, −0.20) was negatively associated with community’s perceptions of the police. No association was observed for gender and race/ethnicity.

**Conclusion:**

A large proportion of Harlem residents have negative perceptions of the police, which are influenced by social and geographical factors. Implementing targeted community programs to build trust and partnerships with law enforcement may be particularly beneficial for economically disadvantaged communities exposed to violence.

## Introduction

Community perceptions of the police are multifaceted, reflecting a dynamic interplay of trust, confidence, and overall satisfaction with law enforcement [1]. Globally, confidence in local police remains relatively high—for example, 84% in Southeast Asia and 76% in North America [2]. However, these aggregate statistics mask significant racial/ethnic and socioeconomic disparities. Understanding how different communities, especially marginalized ones, perceive the police is crucial. Positive perceptions of the police can enhance trust and cooperation with law enforcement [3], while negative views can erode institutional legitimacy and hinder public safety efforts [4]. Moreover, these perceptions also have implications for public health, as distrust in law enforcement is associated with elevated psychological stress and poorer health outcomes in affected communities [5–7].

In the U.S., long-standing patterns of police brutality and racial profiling have contributed to widespread distrust among minoritized communities, particularly Black, Indigenous, and other people of color. This distrust existed well before the COVID-19 pandemic and was exacerbated by high-profile incidents of police violence that sparked national and international outrage [8]. Following the acquittal of Trayvon Martin’s shooter in 2013, the gap between white and Black individuals’ confidence in police widened [9]. Between 2014–2019, confidence in police declined more sharply among Black individuals than their white counterparts, increasing the racial/ethnic disparity—from a 25-point difference to a 30-point difference. George Floyd’s murder during the COVID-19 pandemic further deteriorated confidence in police, reaching unprecedented low levels. According to a 2020 Gallup survey, only 19% of Black respondents and 56% of white respondents expressed “a great deal” or “quite a lot” of confidence in the police. Despite Black individuals’ confidence in police rebounding to 27% in 2021, it remained substantially lower than the 56% reported by white counterparts [10]. Furthermore, Black individuals reported heightened fear and distrust towards law enforcement due to frequent encounters with police, including racial profiling and stops [11–15].

New York City (NYC) provides a unique context for examining police-community relations. A recent study suggested that COVID-19 pandemic-related stay-at-home restrictions were linked to declines in residential burglary, felony assault, grand larceny, sexual violence, and robbery, while protests against police brutality were associated with an increase in felony assault, grand larceny, robbery, and gun-related incidents [16]. Although overall crime trends have fluctuated, recent data suggests a notable increase in low-level arrests, consistent with the enforcement logic of “Broken Windows” policing. Between 2021 and 2023, arrests increased by over 42%, with 88.5% of those arrested for misdemeanors being among New Yorkers of color [17]. This pattern raises concerns about disproportionate policing in minoritized communities and warrants further scrutiny. Harlem is a historically significant Black community in NYC with deep ties to activism, police reform, and racial justice. It has long experienced over-policing, stop-and-frisk practices, and disproportionate arrests, fueling distrust in law enforcement [18,19]. As one of the most economically disadvantaged neighborhoods in NYC and one of the hardest hit by the COVID-19 pandemic [20], Harlem presents a critical context in which to examine police-community relations, perceptions of safety, and systemic inequality in the post-pandemic era.

Previous research has explored various dimensions of community’s perceptions of police—such as policing and collective efficacy [21], resident evaluations of police performance [22,23], police violence [24], and direct interactions [4]. Current literature often predates the COVID-19 pandemic [22–27] or focuses narrowly on specific contexts, such as traffic and pedestrian stops [28]. Additionally, some studies showed that socioeconomic factors such as younger age, lower social class, and residency in high-crime neighborhoods tend to correlate with less favorable views of the police [4,29–31]. Few have systematically investigated how social factors (e.g., experiencing discrimination and perceptions of community safety) and geographical context (e.g., housing type and district community) intersect to influence these perceptions within Black-majority communities in urban post-pandemic settings [26,27]. Comprehensive investigations of these relationships are necessary to provide a more holistic understanding of public attitudes toward policing.

Guided by the Ecological Systems Theory [32], this study examines two key levels: the microsystem and exosystem. The microsystem focuses on community safety during COVID-19, as a direct influence on perceptions of police via increased social vulnerability, changes in public behavior, and evolving patterns of crime and enforcement [33]. At the exosystem level, we included broader factors like housing and neighborhood characteristics, such as community boards, as they shape local policing policies, resource allocation, and residents’ experiences within distinct administrative boundaries in NYC. Thus, this study aimed to examine the community’s perceptions of the police and its associations with demographics, social (a history of discrimination and community safety during COVID-19), and geographic factors (housing and community boards) among a predominantly Black community in NYC. Specifically, this study sought to answer the following research questions: (1) How do sociodemographic factors—such as age and gender—relate to perceptions of the police among residents of a predominantly Black community in NYC? (2) What is the relationship between social factors, including experiences of discrimination and perceptions of community safety, and views of the police? (3) Are there significant differences in police perceptions across various housing types or community boards within Harlem?

## Materials and methods

### Study design and sample size

This cross-sectional study employed convenience sampling and collected online data via the Qualtrics platform from self-reported Harlem residents aged 18 years or older, from April to September 2021. Participants who did not self-report being Harlem residents or were under 18 years of age were excluded from the study. Consistent with a rigorous verification process described previously [20,34–36], including open-ended queries and honeypot questions—hidden fields created using JavaScript that are invisible to human respondents, but detectable by bots. Furthermore, we conducted comprehensive cross-referencing of responses by comparing information from the completed online questionnaire with data obtained through follow-up emails and phone calls, as well as online tools (e.g., whitepages.com). Of the respondents, 393 participants were eligible for the final analysis, with missing data comprising less than 2% of total responses.

### Measurements

*Community perceptions of the police:* We developed a 10-item questionnaire encompassing aspects of police responsiveness, safety, and racism [34–36]. The questionnaire included five positive statements about the police, such as *“The police are responsive to calls from my community”* or *“I find the police to be helpful in managing crime in my neighborhood;”* and five negative statements, such as “*My community will be safer with fewer police*” or “*The police in the community behave in racist ways.*” Each item was scored on a Likert scale ranging from 1 (Strongly disagree) to 5 (Strongly agree). For negative statements, scores were recoded to ensure consistency. Exploratory factor analysis for 10 items using principal factor, Eigen value, and parallel analyses indicated a unifactorial structure. However, given confirmatory factor analysis and Cronbach alpha, the five negative items were retained as they showed the strongest internal consistency (α = 0.69), with factor loadings ranging from 0.30 to 0.7, and 33.5% of variance explained (S1 Table). Model fit indices suggested a modest fit, with CFI = 0.88, TLI = 0.75, SRMR = 0.07, and RMSEA = 0.158. These five items were used to construct the total score for community perceptions of the police, ranging from 5 to 25, with higher scores indicating a more positive community perception of the police.

*Community safety*: was assessed using a single item inquired participants’ perception of safety in their community following the onset of the COVID-19 pandemic: *“How safe or unsafe you feel in your community after the COVID-19 pandemic began?”* The question was scored on a 5-point Likert scale ranging from 1 (Extremely unsafe) to 5 (Extremely safe).

*A history of discrimination:* We evaluated experiences of discrimination, obstacles, or feeling of inferiority in specific contexts, such as school, work, medical care (both general and COVID-19-related), public spaces, interactions with law enforcement, and other situations. Participants who responded “yes” to any of these contexts were classified as having experienced discrimination [36].

*Sociodemographic characteristics:* Participants were asked to provide the following demographic information [20,34,35]: age in years, gender (male, female, and other), race/ethnicity (African American/Black, Latino/Asian/Multiracial, and white), born outside the U.S. (yes or no), currently married (yes or no), household size (only yourself, yourself and another, or 3–8 people), educational attainment (high school or less, associate’s or college degree, bachelor’s or graduate degree), employment status (unemployed or employed), work changed during COVID-19 (yes or no), and annual income (<$25K, $25K-$49K, ≥ $50K). We also collected geographic information, including housing type (market-rate vs. low-income) and district/community boards (9, 10, and 11).

### Statistical analysis

Mean and standard deviation (SD) were presented for community perception of the police while frequencies and percentages were reported for sociodemographic characteristics. For bivariate analyses, t-tests and one-way analysis of variance were used to examine differences in community’s perception of the police across sociodemographic, social, and geographical factors.

Although Likert-scale items are ordinal, research shows that when using a 5-level (or greater) Likert scale, total scores can be treated as continuous, as they may approximate a normal distribution without affecting the results [37–40]. Normality tests (not shown) indicated our data met assumptions. Thus, multivariable linear regression models were employed to assess the associations of interest with community’s perception of the police. This model was adjusted for age group, gender, race/ethnicity, education, employment status, housing type, community safety, and history of discrimination. Community district was excluded from the adjustment in the final model because it was not significantly associated with the outcome in bivariate analysis and thus has minimal impact on model coefficients.

Beta coefficients (β) and 95% confidence intervals (CI) were reported. Additionally, we conducted sensitivity analyses by repeating the main analyses stratified by racial/ethnic groups, specifically for Black (n = 189), Latino (n = 41), and white adults (n = 138). However, these subgroup analyses should be interpreted with caution because of the relatively small sample sizes within each group, which may limit statistical power and generalizability. Data were cleaned and analyzed using STATA version 18.

### Ethical considerations

This study was reviewed and approved by the Institutional Review Board of the City University of New York’s Graduate School of Public Health and Health Policy. All participants provided an online written consent form before completing the survey.

## Results

### Demographic factors and their association with community’s perception of the police

The mean age was 34.7 (SD = 8.9), with almost half of participants aged 30–39 years old (48.6%). More than half were female (53.7%), and almost half identified as African American/Black (48.1%). Most participants (92.1%) were born in the U.S., and 70% of the participants were married. Participants were more likely to report living with 3–8 people in households (73.0%) and hold at least an associate’s or college degree (72.3%). Approximately 19.1% experienced unemployment and 67.9% had work changes during COVID-19. Participants’ annual income ranged from $25K to $49K (57.4%; Table 1). Participants were more likely to currently reside in market-rate housing (64.6%) and District 10 (55%). More than two-thirds of participants reported experiencing discrimination (67.9%).

**Table 1:** Demographic characteristics and their associations with community’s perception of the police among Harlem residents, New York City: 2021.

	Total n (%)	Perception score Mean±SD	P-value^+^
**Age groups**			<0.001
Mean±SD	34.7 ± 8.9	–	
18-29	110 (28.0)	9.44 ± 3.68	
30-39	191 (48.6)	10.31 ± 3.58	
40-81	92 (23.4)	11.70 ± 3.76	
**Gender**			0.015
Female	211 (53.7)	10.90 ± 4.01	
Male	145 (36.9)	9.82 ± 3.42	
Other	37 (9.4)	9.76 ± 1.88	
**Race/ethnicity**			0.124
African American/Black	189 (48.1)	10.74 ± 4.18	
White	138 (35.1)	10.26 ± 3.17	
Latino/Asian/Multiple races	66 (16.8)	9.68 ± 3.33	
**Born outside the U.S.**			0.457
No	362 (92.1)	10.35 ± 3.69	
Yes	31 (7.9)	10.87 ± 4.15	
**Being currently married**			0.439
No	118 (30.0)	10.17 ± 3.62	
Yes	275 (70.0)	10.49 ± 3.78	
**Household size**			0.052
Only yourself	38 (9.7)	9.84 ± 3.55	
Yourself and another	68 (17.3)	9.54 ± 2.51	
3-8 people	287 (73.0)	10.66 ± 3.95	
**Educational attainment**			<0.001
High school and less	109 (27.7)	11.77 ± 3.66	
Associate’s or college’s degree	205 (52.2)	10.14 ± 3.49	
Bachelor or graduate degree	79 (20.1)	9.14 ± 3.89	
**Respondents’ unemployment status**			<0.001
Employed (Full-time, part-time, etc.)	318 (80.9)	9.66 ± 3.42	
Unemployed	75 (19.1)	13.48 ± 3.42	
**Having work changed during COVID-19**			0.364
No	126 (32.1)	10.14 ± 3.91	
Yes	267 (67.9)	10.51 ± 3.64	
Respondents’ yearly income*			0.137
<$25,000	97 (25.1)	10.57 ± 3.50	
$25,000 to $49,999	222 (57.4)	10.54 ± 3.81	
≥$50,000	68 (17.6)	9.55 ± 3.63	
**Housing type**			0.002
Market rate	254 (64.6)	10.82 ± 3.74	
Low-income	139 (35.4)	9.60 ± 3.58	
**District/Community board**			0.297
Community board 9	136 (34.6)	10.07 ± 3.69	
Community board 10	216 (55.0)	16.66 ± 3.87	
Community board 11	41 (10.4)	10.05 ± 2.98	
**Having ever experienced discrimination**			<0.001
No	126 (32.1)	11.98 ± 4.08	
Yes	267 (67.9)	9.64 ± 3.30	

+ t-test and one-way ANOVA tests; *Refused to answer (n = 6).

In bivariate analyses, factors significantly associated with community perception of the police included age group, gender, educational levels, unemployment status, housing type, and experiences of discrimination (all p-values <0.05).

### Prevalence of community’s perception of the police

Participants were more likely to disagree or strongly disagree with the following positive statements about the police: “*The police are equipped to deal with mental health issues*” (39.2%), and “*The police have been respectful and responsive to the needs of demonstrators*” (36.1%, Table 2). Around a quarter of participants disagreed or strongly disagreed with the statements “*I find the police to be helpful in managing crime in my neighborhood*” (24.7%), “*I feel safer when I see police presence in my community*” (25.2%), and “*The police are responsive to calls from community*” (26.7%).

**Table 2:** Community’s perception of the police among Harlem residents, New York City: 2021.

#	Statements	% strongly disagree/disagree	% neither agree nor disagree	% strongly agree/agree
1	The police are responsive to calls from my community	105 (26.7)	115 (29.3)	173 (44.0)
2	I feel safer when I see police presence in my community	99 (25.2)	91 (23.2)	203 (51.6)
3	I find the police to be helpful in managing crime in my neighborhood	97 (24.7)	154 (39.2)	142 (36.1)
4	The police are equipped to deal with mental health issues	154 (39.2)	137 (34.9)	102 (25.9)
5	The police have been respectful and responsive to the needs of demonstrators	142 (36.1)	109 (27.8)	142 (36.1)
6	I feel unsafe when I interact with the police	157 (40.0)	125 (31.8)	111 (28.2)
7	My community will be safer with fewer police	161 (41.0)	124 (31.5)	108 (27.5)
8	The police in my community behave in racist ways	117 (29.8)	138 (35.1)	138 (35.1)
9	Someone in my household was unfairly stopped by the police	177 (45.0)	93 (23.7)	123 (31.3)
10	I feel safer with fewer police	124 (31.5)	154 (39.2)	115 (29.3)

Items 6–10 constructed the total score for community perceptions of the police (Mean = 10.33, SD = 3.73).

The proportions of residents who agreed or strongly agreed with the negative statements ranged from 27.5% (“*My community will be safer with fewer police*”) to 35.1% (“*The police in my community behave in racist ways*.” Around 30% strongly agreed or agreed with the statements, “*Someone in my household was unfairly stopped by the police*” (31.9%) and “*I feel safer with fewer police*” (29.3%).

### Associations between sociodemographics, social and geographical factors, and community’s perception of the police

In multivariable linear regression (Table 3), a more negative perception of the police was observed among adults aged 18−29 years (β = −2.45, 95%CI: −3.61, −1.28) and 30−39 years (β = −1.80, 95%CI: −2.82, −0.77) compared with individuals aged 40−81 years old. Higher levels of education (Associate’s or college degree: β = −0.83, 95%CI: −1.77, −0.10; and bachelor’s or graduate degree: β = −2.10, 95%CI: −3.15, −1.05) were significantly associated with more negative police perception relative to individuals with higher school degree or less, whereas being employed was associated with less favorable police community perception (β = −2.15, 95%CI: −3.32, −0.99). Compared to those who resided in market-rate housing, low-income housing residents had 0.87 (95%CI: −1.62, −0.12) points lower in police perception score. Likewise, those who experienced discrimination had a 1.94-point lower score in community perceptions of the police (β = −1.94, 95%CI: −2.72, −1.16). Higher levels of community safety during COVID-19 were associated with more negative police perception (β = −0.63, 95%CI: −1.05, −0.20). No association was found for gender and race/ethnicity with community’s perception of the police.

**Table 3:** Adjusted beta coefficients (β)* and their 95% confidence intervals (CI) for the association of sociodemographic characteristics with community’s perception of the police among Harlem’s residents, New York City: 2021.

	β	95% CI
**Age group** (vs. 40–81 years old)		
18-29 years old	−2.45	−3.61, −1.28
30-39 years old	−1.80	−2.82, −0.77
**Being male** (vs. female)	0.13	−0.69, 0.95
**Race/ethnicity** (vs. white)		
African American/Black	−0.59	−1.44, 0.26
Latino/Asian/Multiple races	−0.97	−2.05, 0.12
**Educational level** (vs. high school and less)		
Associate’s or college’s degree	−0.83	−1.77, −0.10
Bachelor or graduate degree	−2.10	−3.15, −1.05
**Being employed** (vs. unemployed)	−2.15	−3.32, −0.99
**Living in low-income housing** (vs. market-rate housing)	−0.87	−1.62, −0.12
**Community safety during COVID-19**	−0.63	−1.05, −0.20
**A history of discrimination** (vs. no)	−1.94	−2.72, −1.16

*Coefficients are mutually adjusted for variables included in the model.

In the subgroup analysis, the adjusted analyses for Black adults mirrored the findings from the overall sample, with younger age, higher education, employment, residence in low-income housing, higher levels of community safety during COVID-19, and a history of discrimination all associated with a more negative perception of the police (S2 Table). Among Latino adults, only community safety was significantly associated with a more negative perception of the police (β = −1.44, 95%CI: −2.74, −0.14). Among white adults, only individuals aged 30−39 (β = −2.49, 95%CI: −4.79, −0.20) were associated with a more negative perception of the police.

## Discussion

Our findings indicate negative perceptions of police across several dimensions, including their handling of mental health issues, behavior towards demonstrators, responsiveness to incidents, and overall effectiveness in maintaining public safety. Previous research has emphasized the lack of necessary training and expertise among police officers to effectively identify and respond to mental health crises. In particular, the challenges of differentiating between mental health emergencies and other health crises (e.g., drug overdose, hypoglycemic shock, or epileptic seizures) were mentioned [41,42]. Given that police officers are often the first responders in such situations [43], our findings highlight the need for enhanced mental health training and protocols that enable officers to not only respond effectively, but also connect individuals to appropriate mental health services. Improving police capacity in this area could reduce harmful outcomes, alleviate community tensions, and foster trust between the police and community, both during and beyond the pandemic.

Additionally, approximately 30% of participants expressed agreement with statements indicating feelings of unsafety during police interactions, believing that the community would be safer with fewer police, perceiving racism among police, and experiencing unfair police stops. These feelings of fear and distrust towards law enforcement can be a significant driver of public health outcomes in racialized risk environments [11]. Within Black communities, police encounters encompass various types of interactions such as racial profiling, stop-and-frisk, arrests, and aggressive policing [12]. Additionally, individuals from minoritized backgrounds reported more frequent encounters with the police than white people [13]. For instance, a study conducted in Washington, DC, found that despite comprising only 47% of the population, Black residents accounted for 83% of stop-and-frisk searches [14]. Addressing issues related to trust, fairness, and potential biases within the community-police relationship is necessary. Proactive efforts are necessary to promote equitable and unbiased policing practices, while also exploring alternative approaches to community safety. Additionally, to complement our quantitative approach, employing a qualitative design would be beneficial since such a design can provide in-depth insights into the lived experiences of community members, their interactions with the police, and the contextual factors influencing their perceptions.

This study further identifies sociodemographic factors influencing police perceptions. Younger residents and those residing in low-income housing had a lower perception of law enforcement. These findings corroborate prior research indicating that factors such as younger age, lower social class, and residing in economically challenged neighborhoods with high crime rates were associated with less favorable attitudes about the police [15,29–31]. Moreover, individuals with higher education and employed had lower perceptions of the police, potentially due to increased awareness of social inequities, political engagement, and an understanding of criticisms of the police [34,44]. This emphasizes the need for a nuanced exploration of how sociodemographic factors intersect with community perceptions of law enforcement, thereby offering valuable insights for targeted interventions and policy development. Notably, our study did not observe any significant associations of gender and race/ethnicity with the community’s perception of the police. This lack of association may be attributed to the notion that socioeconomic status may outweigh race in determining attitudes toward the police, and racial differences may diminish after accounting for social class [29,31]. Furthermore, the combination of race and ethnicity, as well as sex and gender, in the same question may have contributed to this lack of association. Future research should separate them to identify their specific impacts.

We observed that individuals who reported higher levels of perceived community safety were less likely to express better perceptions of police. This finding contrasts with a previous study indicating that residents who perceived their neighborhoods as safe and felt satisfied with overall safety tended to hold a more favorable opinion of police, regardless of individual characteristics such as age and race/ethnicity [45]. One possible explanation for this discrepancy could be the definition of community safety. In our study, we used a single item to assess community safety without providing additional context, which may have led to varying interpretations among participants. It is also plausible that the perceived safety of a community is influenced by factors unrelated to policing, such as strong social bonds and low crime rates. Consequently, residents may view police presence as being less necessary in such contexts. Conversely, those who reported experiencing discrimination were negatively associated with police perception. Negative encounters with discrimination may lead to diminished trust or confidence in law enforcement agencies [11,46]. A closer look at the data revealed that most of these discriminatory experiences occurred on the street or in a public setting (64.5%). Therefore, addressing discrimination within these environments is crucial not only for social justice, but also for fostering positive police-community relations and improving perceptions of law enforcement.

Overall, this study contributes to the literature by highlighting how structural and contextual factors—including socioeconomic status, housing, discrimination, and community cohesion—shape police perceptions in a historically marginalized urban setting, especially in the post-COVID-19 period. The findings challenge assumptions about the role of police in ensuring safety and underscore the need for alternative community-centered safety strategies. Policymakers, practitioners, and researchers must prioritize these insights to develop equitable policing reforms and interventions that genuinely address community needs and foster trust. Our study had several limitations that warrant discussion. Despite collaborating with experts in the field, the developed scale consisting of 10 items to measure community policing perceptions exhibited a low Cronbach’s alpha of 0.41 [34–36,47]. Although we conducted factor analysis to identify correlated items to maintain five items with a Cronbach’s alpha of 0.69 in the final analysis, it is possible that these five items may not fully capture the underlying construct. Thus, further investigation (e.g., predictive or concurrent validity) is necessary to fully comprehend its psychometric properties. Additionally, the measurement of community safety relied on a single item and the categorization of sex/gender and race/ethnicity was combined, thereby warranting careful consideration and additional scrutiny. It is important to note that this study primarily concentrated on a predominantly Black community in NYC; hence, its findings may not reflect the broader demographics of the entire city. Finally, due to small sample sizes, Latino, Asian/Asian American, and multiracial/other participants were combined into a single “other minoritized group” for analysis. Although necessary for statistical power, this approach may mask important differences between these groups. Future studies should aim for larger and more diverse samples to explore these distinctions.

## Conclusion

This study revealed negative perceptions of police responsiveness, community safety, and officer behavior among Harlem residents. The major implications of our findings are that police reform must specifically address training in mental health crisis responses and actively combat discriminatory practices to rebuild trust within minoritized communities. Notably, younger residents, those with lower socioeconomic status, and those who experienced discrimination were more likely to hold negative views. The counterintuitive association between perceived safety and lower police perception deserves further exploration, as it suggests a nuanced understanding of “safety” in these communities that may not always align with traditional notions of police presence. To improve police-community relations, implementing programs that address the needs of marginalized and disadvantaged communities within Harlem could be beneficial. Additionally, further research is necessary to understand how specific policing practices impact community dynamics, and consequently, residents’ perceptions of the police. Furthermore, a key limitation of this study is that the construct measuring community’s perceptions of police has not yet been fully validated, highlighting the need for additional qualitative research to deepen understanding and improve measurement of this construct. Addressing these limitations will strengthen future studies and contribute to more effective interventions.

## Supporting information

S1 Table Factor analysis for community’s perceptions of the police in the community among Harlem’s residents, New York City: 2021. (DOCX)

S2 Table Adjusted beta coefficients (β)* and their 95% confidence intervals (CI) for the association of sociodemographic characteristics with community’s perception of the police stratified by race/ethnicity among Harlem’s residents, New York City: 2021. (DOCX)
