# Supplementary material for: Community’s perceptions of the police during COVID-19 in Harlem, New York, a predominantly Black community: Social and geographical dimensions
Source: PLoS One. 2025 Oct 6;20(10):e0329027. doi: 10.1371/journal.pone.0329027 (PMC12500094; doi:10.1371/journal.pone.0329027)
Supplement: S1 Table — (DOCX) [file pone.0329027.s001.docx]

**S1 Table.** Factor analysis for community’s perceptions of the police in the community among Harlem’s residents, New York City: 2021

|  |  | **Mean** | **Skewness** | **Kurtosis** | **Loading factor for EFA** | **Loading factor for CFA** | **α if deleted for CFA** |
| --- | --- | --- | --- | --- | --- | --- | --- |
| 1 | The police are responsive to calls from my community | 3.21 | -0.18 | 2.26 | -0.11 | - | - |
| 2 | I feel safer when I see police presence in my community | 3.36 | -0.37 | 2.24 | -0.09 | - | - |
| 3 | I find the police to be helpful in managing crime in my neighborhood | 3.13 | -0.17 | 2.58 | 0.39 | - | - |
| 4 | The police are equipped to deal with mental health issues | 2.78 | 0.11 | 2.26 | 0.38 | - | - |
| 5 | The police have been respectful and responsive to the needs of demonstrators | 3.01 | 0.01 | 2.13 | 0.46 | - | - |
| 6 | I feel unsafe when I interact with the police | 2.85 | 0.15 | 2.33 | 0.68 | 0.75 | 0.58 |
| 7 | My community will be safer with fewer police | 2.87 | 0.27 | 2.40 | 0.64 | 0.66 | 0.65 |
| 8 | The police in my community behave in racist ways | 3.08 | -0.05 | 2.35 | 0.55 | 0.48 | 0.65 |
| 9 | Someone in my household was unfairly stopped by the police | 2.79 | 0.18 | 1.99 | 0.32 | 0.30 | 0.73 |
| 10 | I feel safer with fewer police | 3.02 | 0.18 | 2.50 | 0.63 | 0.66 | 0.60 |
