# Supplementary material for: Community’s perceptions of the police during COVID-19 in Harlem, New York, a predominantly Black community: Social and geographical dimensions
Source: PLoS One. 2025 Oct 6;20(10):e0329027. doi: 10.1371/journal.pone.0329027 (PMC12500094; doi:10.1371/journal.pone.0329027)
Supplement: S2 Table — (DOCX) [file pone.0329027.s002.docx]

**S2 Table.** Adjusted beta coefficients (β)* and their 95% confidence intervals (CI) for the association of socio-demographic characteristics with community’s perception of the police stratified by race/ethnicity among Harlem’s residents, New York City: 2021

|  | **β** | **95% CI** |
| --- | --- | --- |
| **Black adults (n=189)** | | |
| **Age group** (ref=40-81) |  |  |
| 18-29 years old | -2.61 | -4.08, -1.14 |
| 30-39 years old | -1.32 | -2.49, -0.14 |
| **Being male** (vs. female) | 0.20 | -0.98, 1.38 |
| **Educational level** (vs. High school and less) |  |  |
| Associate’s or college’s degree | 0.26 | -0.82, 1.33 |
| Bachelor or graduate degree | -1.96 | -3.37, -0.56 |
| **Being employed** (vs. unemployed) | -2.29 | -3.75, -0.84 |
| **Living in low-income housing** (vs. Market-rate housing) | -1.53 | -2.51, -0.55 |
| **Community safety during COVID-19** | -1.02 | -1.56, -0.48 |
| **A history of discrimination** (vs. no) | -3.05 | -4.08, -2.01 |
| **Latino adults (n=41)** | | |
| **Age group** (ref=40-81) |  |  |
| 18-29 years old | -1.12 | -5.31, 3.07 |
| 30-39 years old | -1.44 | -5.38, 2.50 |
| **Being male** (vs. female) | -2.75 | -5.73, 0.24 |
| **Educational level** (vs. High school and less) |  |  |
| **Associate’s or college’s degree** | 0.99 | -1.94, 3.93 |
| **Bachelor or graduate degree** | -1.56 | -5.38, 2.27 |
| **Being employed** (vs. unemployed) | 2.69 | -0.34, 5.73 |
| **Living in low-income housing** (vs. Market-rate housing) | -0.35 | -2.97, 2.27 |
| **Community safety during COVID-19** | -1.44 | -2.75, -0.14 |
| **A history of discrimination** (vs. no) | 0.90 | -1.78, 3.57 |
| **White adults (n=138)** | | |
| **Age group** (ref=40-81) |  |  |
| 18-29 years old | -1.88 | -4.35, 0.59 |
| 30-39 years old | -2.49 | -4.79, -0.20 |
| **Being male** (vs. female) | 0.65 | -0.65, 1.95 |
| **Educational level** (vs. High school and less) |  |  |
| Associate’s or college’s degree | -1.07 | -2.86, 0.73 |
| Bachelor or graduate degree | 0.08 | -2.12, 2.29 |
| **Being employed** (vs. unemployed) | -5.46 | -12.42, 1.50 |
| **Living in low-income housing** (vs. Market-rate housing) | 0.42 | -0.94, 1.78 |
| **Community safety during COVID-19** | 0.28 | -0.54, 1.10 |
| **A history of discrimination** (vs. no) | 0.14 | -1.23, 1.51 |

*Coefficients are mutually adjusted for variables included in the models.
